# 

**Published:** 1911-04

**Authors:** 


### CONTENTS.

ORIGINAL COMMUNICATIONS—

Mayfield-Tyzzer Hospital for Men at Laichon Fu, China. By J. M. Gaston .......... 1
The Social Evil—Its Prevalence and Results. By R. R. Kime .......... 6
Poliomyelitis; Apropos of the Spread Southward of the Disease; Recent Researches; Fallacies in Diagnosis; Misconceptions about Treatment. By Tom A. Williams, Washington, D. C. .......... 10
Obligations of the Medical Profession. By Frances Bradley .......... 25
Treatment of Anterior Poliomyelitis. By Theo Toepel, M. D., Atlanta, Ga. .......... 29
The Secret Commission Evil. By J. P. Lord, Omaha .......... 31
Editorials .......... 40
News and Notes .......... 42
Book Reviews .......... 52

